# Noninvasive Preimplantation Genetic Testing in Recurrent Pregnancy Loss and Implantation Failure: Breakthrough or Overpromise?

**DOI:** 10.3390/cells14201591

**Published:** 2025-10-14

**Authors:** Grzegorz Mrugacz, Aleksandra Mospinek, Joanna Głowacka, Oskar Sprawski, Lidia Kawałek, Wiktoria Gąsior, Julita Machałowska, Yekaterina Sidorova, Patrycja Borecka, Aleksandra Bojanowska, Weronika Szczepańska

**Affiliations:** 1 Center for Reproductive Medicine Bocian, Białystok Branch, 26 Akademicka St., 15-267 Białystok, Poland; gmrugacz@klinikabocian.pl (G.M.);; 2 Center for Reproductive Medicine Bocian, Łódź Branch, 157a Piotrkowska St., 90-440 Łódź, Poland; 3 Center for Reproductive Medicine Bocian, Poznań Branch, 77a Dąbrowskiego St., 60-529 Poznań, Poland; 4 Center for Reproductive Medicine Bocian, Lublin Branch, 26 Relaksowa St., 20-819 Lublin, Poland; 5 Center for Reproductive Medicine Bocian, Szczecin Branch, 1 Brama Portowa Sq, 70-225 Szczecin, Poland; 6 Center for Reproductive Medicine Bocian, Gdańsk Branch, 103a Grunwaldzka Ave, 80-236 Gdańsk, Poland

**Keywords:** noninvasive preimplantation genetic testing (niPGT), recurrent pregnancy loss (RPL), recurrent implantation failure (RIF), embryonic aneuploidy, trophectoderm biopsy, preimplantation genetic testing for aneuploidy (PGT-A), in vitro fertilization (IVF)

## Abstract

**Highlights:**

**What are the main findings?**

- Current evidence indicates noninvasive PGT-A (niPGT-A) has variable and suboptimal diagnostic accuracy compared to trophectoderm biopsy.
- There is a definitive lack of robust, prospective trial data demonstrating that niPGT-A improves live birth rates, specifically for patients with recurrent pregnancy loss (RPL) or implantation failure (RIF).

**What is the implication of the main finding?**

- niPGT-A is not yet reliable enough to replace invasive PGT-A as the standard of care for managing RPL and RIF. It should only be considered an investigational tool.
- The responsible integration of niPGT-A into clinical practice requires rigorous multicenter RCTs, international protocol standardization, and validation within high-risk RPL/RIF populations.

**Abstract:**

**Background**: Recurrent pregnancy loss (RPL) and recurrent implantation failure (RIF) are significant challenges in reproductive medicine. For both, embryonic aneuploidy is the leading etiological factor. Preimplantation genetic testing for aneuploidy (PGT-A) via trophectoderm biopsy is the current standard for embryo selection. However, it is limited by its invasiveness, potential for embryo damage, and diagnostic errors due to mosaicism. **Rationale/Objectives**: This review critically evaluates the emerging role of noninvasive PGT (niPGT). NiPGT analyzes cell-free DNA from spent blastocyst culture media, thus, it is a potential alternative for managing RPL and RIF. Hence, the primary objective is to determine whether current evidence supports niPGT as a reliable replacement for conventional biopsy-based PGT-A in these high-risk populations. **Outcomes**: The analysis reveals that niPGT offers significant theoretical advantages. These include complete non-invasiveness, enhanced embryo preservation, and high patient acceptability. However, its clinical application is hampered by substantial limitations. Key amongst them is the inconsistent and often suboptimal diagnostic accuracy (sensitivity 70–85%, specificity 88–92%) compared to biopsy. Other significant factors include the high rates of amplification failure (10–50%), vulnerability to maternal DNA contamination, as well as low DNA yield. Crucially, there is a definitive lack of robust, prospective randomized controlled trial (RCT) data demonstrating improved live birth rates or reduced miscarriage rates specifically in RPL and RIF cohorts. As such, niPGT is not yet ready to be a standalone clinical adoption in RPL and RIF cases. However, it may serve as a valuable adjunct for rescue scenarios following biopsy failure or for ethical reasons. **Wider Implications**: The integration of niPGT with artificial intelligence, time-lapse imaging, and multi-omics profiling underlies a promising future. However, its transition from a predominantly research tool to a clinical standard necessitates various critical undertakings. These include rigorous multicenter RCTs, standardizing international protocol, and tailoring validation for the RPL and RIF subgroups. This review highlights the need for cautious optimism, positing that evidence-based integration, rather than premature adoption, is essential to realizing niPGT’s full potential without compromising patient care in these complex fertility scenarios.

## 1. Introduction

Recurrent pregnancy loss (RPL) and recurrent implantation failure (RIF) are representative of the significant challenges in reproductive medicine that many face [1,2]. These two conditions foster significant clinical and emotional burdens [2]. RPL, from a layman’s point of view, refers to 2 or more consecutive pregnancy losses before 20 weeks [1,2]. On the other hand, RIF is hallmarked by multiple failed IVF cycles despite high-quality embryo transfers [1,2]. These two reproductive health complications frequently stem from multiple causes. However, embryonic aneuploidy is highlighted as the predominant contributor, more so in advanced maternal age and idiopathic cases [2,3].

Preimplantation genetic testing for aneuploidy (PGT-A) is the current gold standard via trophectoderm biopsy [3,4,5,6]. Its central aim is to optimize embryo selection and reduce miscarriage risks [7]. However, its invasive nature raises concerns about embryo integrity, diagnostic inaccuracies due to mosaicism, and ethical dilemmas [6,8]. These limitations have fostered and accelerated the development of noninvasive PGT (niPGT) [9,10,11]. NiPGT analyzes cell-free DNA (cfDNA) in spent blastocyst culture media [9]. Existing evidence in the form of early studies suggests that niPGT has the potential to mirror PGT-A in detecting aneuploidies [9,10,11]. However, critical technical hurdles persist. These include contamination risks, inconsistent DNA yield, and insufficient validation in high-risk populations like RPL/RIF [11].

This review critically evaluates niPGT’s viability as a clinical tool for RPL/RIF management, arguing that despite its promise as a breakthrough, current evidence supports only cautious integration into IVF practice. Hence, thorough standardization and rigorous validation ought to be upheld before fully replacing invasive methods. This review begins by assessing the pathophysiology of RPL/RIF and aneuploidy’s role, then advancing to the limitations of conventional PGT-A. NiPGT will then be addressed as the potential savior, specifically covering its methodological advances and reliability concerns, as well as the clinical evidence for niPGT in RPL/RIF cohorts. The review will then conclude by highlighting future directions integrating AI, epigenetics, and multicenter trials.

## 2. Understanding RPL and RIF: Clinical Definitions and Burden

RPL and RIF are complex conditions with overlapping and distinct etiologies. They are summarized in Table 1 below.

### 2.1. The Challenge of Idiopathic Cases

Extensive workups have been fostered. However, 40–50% of RPL and approximately 30% of RIF cases remain idiopathic [13,14]. This diagnostic gap can be attributed to various factors:

- Undetectable molecular defects—This is evidenced by subtle endometrial transcriptomic dysregulation, such as HOXA10 downregulation or sperm DNA fragmentation [13,32].
- Microbiota imbalances—Hallmarked by altered endometrial Lactobacillus dominance that correlates with RPL [20,33].
- Significant limitations in testing—Standard panels often omit immune/metabolic biomarkers like ANXA5 or NK cell functional assays, resulting in inevitable discrepancy [26,29].

### 2.2. Psychological and Financial Toll

RPL and RIF have significant effects on patients.

- Psychological—Patients with RPL/RIF experience 3 times higher rates of depression and anxiety [17,21]. Many also experience grief and stigma. For instance, 68% of those affected report strained relationships and social isolation [12,17].
- Financial burden—A single IVF cycle averages $12,000–$15,000 while cumulative RIF treatments often exceed $50,000 [12,16,21]. There is also lost productivity. RPL-associated absenteeism, for instance, is approximated to cost each patient $6000–10,000 annually in lost productivity [17,34].

## 3. The Role of Embryonic Aneuploidy in RPL and RIF

### 3.1. Understanding Aneuploidy: Definition and Types

Embryonic aneuploidy is the primary biological cause of RPL and RIF. It encompasses several distinct types of chromosomal abnormalities, each with specific clinical implications. They are detailed in Table 2 below.

### 3.2. Aneuploidy as a Primary Cause of Implantation Failure and Miscarriage

In RIF, aneuploidy is the predominant cause, with 60–70% of high-quality morphologic embryos being chromosomally abnormal [43,47,48]. Regarding implantation failure, aneuploid embryos exhibit significantly lower implantation rates than euploid counterparts. For example, clinical studies show a 3.7-fold reduction [40,42]. This disparity is biologically rooted in the widespread gene expression imbalances caused by chromosomal abnormalities, which disrupt key processes essential for implantation, such as trophoblast invasion and development [2]. As such, selecting euploid embryos via PGT-A significantly improves implantation and live birth rates while reducing miscarriage rates per transfer for RIF patients [4,40,42,43,44,48,49]. However, the benefit of PGT-A on cumulative live birth rates, particularly in younger women with RIF, remains debated. Some studies show no significant improvement over untested embryo transfer [41,47].

In RPL, aneuploidy is implicated in 50–70% of cases, rising to over 80% in women over 40 [31,35]. Even after implantation, it causes approximately 60% of first-trimester miscarriages [45,46]. While RPL patients may have a similar overall aneuploidy rate to other infertility patients, some evidence suggests a higher prevalence in those with a history of prior aneuploid losses [35]. The mechanisms by which aneuploidy leads to miscarriage include placental insufficiency, such as trisomy 16 disrupting trophoblast function and direct cell cycle arrest triggered by monosomies [35,36].

### 3.3. Impact of Maternal Age and Diminished Ovarian Reserve (DOR)

Maternal age influences aneuploidy rates, which rise significantly from approximately 30% at age 30 to 80% by age 40 [4,35,36,38,40,41,42,43,44,45,46,50]. This age-related decline in oocyte quality compromises chromosomal segregation fidelity. Consequently, women aged 35 and above with RPL face a 2.5-fold higher aneuploidy risk, and the prevalence of both RPL and RIF increases with maternal age. This makes PGT-A a more sufficient intervention in the older patients [35,36,38,40,42,43,44,49].

DOR, characterized by reduced oocyte quantity/quality, is an independent risk factor for aneuploidy. A low AMH level (<1.1 ng/mL) is associated with a 45% aneuploidy rate. This is significantly higher than the 28% rate seen with a normal ovarian reserve [38,40,41,43,44,46]. Younger women with DOR can thus paradoxically face aneuploidy risks comparable to older women, a phenomenon attributed to DOR-related mitochondrial dysfunction and increased oxidative stress that promotes meiotic errors [38,51].

### 3.4. Current Embryo Selection Strategies

Table 3 below presents a summative overview of the current embryo selction strategies, categorically highlighting their mode of operation, strengths and key limitations. Covered are the three major approaches curently being used globally.

## 4. Invasive PGT-A: Current Standard and Its Limitations

PGT-A is a significant advancement in assisted reproductive technology (ART), more so in embryo selection. It aims to improve outcomes for patients experiencing RPL and RIF by selecting euploid embryos for transfer. However, its invasive nature and associated limitations warrant further critical evaluation.

### 4.1. Background—Purpose and Types

The primary objective of PGT-A is to identify embryos with the correct number of chromosomes (euploidy), screening out those with missing or having extra chromosomes (aneuploidy). This selection increases implantation rates per embryo transfer, reduces the rate of clinical miscarriage, decreases the time to achieve a live birth by minimizing transfers of embryos with low developmental potential, and fosters single embryo transfer (SET), thus reducing the risk of multiple pregnancies [3,6,39,52,53,54,55,56]. PGT encompasses several types. These include:

- PGT-M (Monogenic disorders)—tests for specific single-gene defects.
- PGT-SR (Structural Rearrangements)—tests for balanced chromosomal rearrangements in carriers such as translocations [39,53].
- PGT-A (Aneuploidy Screening)—screens for numerical chromosomal abnormalities across all 24 chromosomes [6,39,53,55,56]. This is the most commonly used in contexts like RPL, RIF, and advanced maternal age [6,39,54,55,56].

### 4.2. Common Techniques: Trophectoderm Biopsy and Blastocyst Stage Analysis

The 2 significant techniques are Trophectoderm (TE) Biopsy and Blastocyst Stage Analysis. Despite their variances, they all begin with blastocyst culture. With this, embryos are cultured in vitro for 5–6 days post-fertilization. This is to enable them to reach the blastocyst stage, which is characterized by an inner cell mass (ICM) that becomes the fetus and TE that becomes the placenta [6,39,53,54,55]. TE biopsy, the current gold standard, begins with embryo preparation. This entails making a small opening in the zona pellucida, the outer shell, on day 3 or 5, often using laser ablation. The biopsy procedure is then performed on day 5 or 6, where approximately 5–10 cells are gently aspirated and removed from the TE layer [3,6,39,53,54,55,56,57]. This is usually achieved using micromanipulation techniques. The biopsied blastocyst is then cryopreserved while genetic analysis, that is, blastocyst stage analysis, is performed [3,6,39,54,55]. During genetic analysis, the biopsied TE cells undergo DNA amplification through Whole Genome Amplification (WGA). This is then followed by chromosomal analysis, which is conducted by primarily using Next-Generation Sequencing (NGS), to detect aneuploidy and mosaicism [6,8,39,53,54,55,57]. Quantitative PCR (qPCR) can also be leveraged for rapid aneuploidy screening [39].

Compared to cleavage biopsy, TE biopsy has its own superiority and benefits. It is preferred because it removes cells destined for the placenta. This theoretically minimizes the impact on fetal development. As per evidence, the lower embryo damage risk is 2–4% compared to the 15% in cleavage biopsy [54]. Further, with TE, more cells are obtained, hence higher DNA yield, which significantly improves diagnostic reliability, an attribute evidenced by the 99% amplification success. On top of these, blastocyst formation itself is a selection step for developmental efficiency [6,39,53,54,55,57]. TE biopsy also allows for cryopreservation of the biopsied embryo. This ensures that the biopsy procedure is separated from the transfer cycle [3,55].

### 4.3. Benefits: Improved Selection and Reduced Miscarriage Rates

TE biopsy-based PGT-A fosters several significant benefits. These include improved embryo selection. By identifying euploid embryos, PGT-A bypasses aneuploid embryos that are highly likely to fail or miscarry [6,39,52,54,55,56]. Evidence indicates that euploid embryos show 2.5 times higher implantation rates compared to the untested morphologically good embryos in RIF patients [3,59]. Secondly, PGT-A reduces miscarriage rates. Some studies show that in women 35 years and above with RPL, PGT-A reduces the miscarriage risk by 50% [3,55]. This is attributed to avoiding aneuploidy, a leading cause of miscarriage [3,52,55,56]. Further, in their systematic review and meta-analysis, Kasaven et al. (2023) found that PGT-A is associated with a higher live birth rate per embryo transfer [52]. Thirdly, PGT-A increases implantation rates (IR) per transfer. This is because transferring screened euploid embryos generally results in higher IR per transfer compared to when the embryos are transferred without screening [52,55]. Compared to conventional IVF, where 3 or more cycles are required, a single euploid transfer achieves pregnancy in 1–2 cycles [6,55]. Fourthly, PGT-A underlies better prognosis and management of patient expectations [55].

### 4.4. Limitations

Despite its benefits and status as the gold standard, invasive PGT-A exhibits significant limitations. Firstly, with its invasive nature, the risk of embryo damage is high. This is because the biopsy procedure physically removes cells from the embryo. Concerns persist regarding potential short-term impacts, such as the reduced developmental potential post-biopsy, and long-term impacts, specifically subtle developmental effects. Regardless, robust evidence for major harm in live births is still limited [6,39,53,54,55,56,57]. Further, due to the high technical expertise required, poor technique can damage the ICM or compromise embryo viability [53,54,55]. Existing evidence indicates that double biopsies done for confirmation testing lower live birth rates by 39% [54]. Still on embryo damage, double handling of combining biopsy and vitrification subjects the embryo to two potentially stressful procedures [3,54,55,57].

Besides embryo damage risk, another significant limitation is mosaicism and the high potential for false negatives or positives. Also referred to as biological limitation in layman’s language, chromosomal mosaicism, that is, the presence of both euploid and aneuploid cells, is common in human blastocysts. TE biopsy operates by sampling only a few cells from one part of the trophectoderm. This is not fully representative of the chromosomal status of the entire embryo or the ICM [8,39,53,54,55,56]. The risk of false negatives is high, and this can be evidenced by a euploid TE biopsy result masking an aneuploid ICM, hence the false negative effect. This falseness results in the transfer of an abnormal embryo that miscarries or results in an affected live birth [8,53,54,55,56]. Conversely, in the case of a false positive, an aneuploid TE biopsy result tends to misrepresent a mosaic or euploid ICM. The outcome of this is the discarding of a viable embryo [3,8,39,53,54,55,56]. Linked to these false outcomes is diagnostic uncertainty. In their systematic review and meta-analysis, Chen et al. (2025) note limitations in diagnostic accuracy [8]. Further, Viville and Aboulghar (2025) posit that the inherent mosaicism and technical limitations insinuate that PGT-A results are not a definitive diagnosis of the embryo’s true chromosomal profile [56].

The third significant limitation is cost and logistical complexity. While IVF is already expensive, PGT-A adds substantial expenses to it in the form of biopsy procedure fees, genetic analysis costs, and embryo culture and cryopreservation-related costs. Based on existing literature, the cumulative costs for RIF patients exceed $50,000 [55]. This is because the procedure adds $3000–$5000 per cycle to IVF costs, totaling about $15,000–$20,000 for most patients [55,56]. These fees create financial barriers, raising pertinent cost-effectiveness questions, especially in younger patients or those with few embryos [6,39,53,54,55,56]. Another cost issue is evidenced in the form of laboratory infrastructure. PGT-A requires specialized embryology skills, advanced genetic laboratory capabilities (NGS), and robust cryopreservation programs. All of these are costly, making the services exclusive only for the few who can afford [39,54,55]. Expenses are also incurred in terms of cycle management. The procedure requires a freeze-all strategy. This delays the transfer and increases the number of patient visits and overall cycle time, resulting in extra expenses for the patient [6,54,55].

The fourth and last significant limitation is evident in the form of ethical and emotional concerns for patients. Firstly, there is a potential for discarding embryos based on biopsy results that may be inaccurate. More so for mosaic embryos. This raises significant ethical dilemmas for both patients and clinicians [3,54,55,56]. In their study, Morales (2024) highlights this as a major controversy [55]. Secondly, the psychological burden is significant. The evident procedural complexity, cost, waiting periods for results, and the possible likelihood of having no transferable embryos can create significant emotional stress for patients. This is on top of them experiencing infertility or loss [54,55,56]. It is indicated that 52% of patients report anxiety over no transferable embryos after PGT-A [52,55]. The third aspect is the issue of imperfect embryos. The possibility of mosaicism often places patients in difficult decision-making positions. This is regarding the transfer of embryos that experts have labeled as abnormal or intermediate, often with limited robust outcome data [54,55,56]. Last is the aspect of overpromise and expectation management. Concerns exist that PGT-A may be oversold as a guarantee of success. This potentially underlies unrealistic expectations and significant disappointment if cycles fail despite euploid transfers [55,56]. To mitigate this, Kakourou et al. (2024), in their overview, emphasize the importance of acknowledging the limitations of both biology and technology [54].

### 4.5. Summary

Based on the above evidence, TE biopsy-based PGT-A is the established method for assessing embryonic aneuploidy. It offers potential benefits in selecting euploid embryos, reducing miscarriage rates per transfer, and improving implantation rates per transfer, particularly in RPL and AMA [52,55]. However, its invasive nature raises significant fundamental concerns. This includes issues about embryo safety and diagnostic accuracy, caused by embryonic mosaicism, hence false results [3,8,53,54,56]. It also imposes significant financial, logistical, and ethical burdens on patients and clinics [39,54,55,56]. These substantial limitations necessitate the need for continued refinement and rigorous counseling on realistic expectations. They also warrant the pursuit of less invasive and more reliable alternatives like noninvasive PGT (niPGT) [57].

## 5. Noninvasive PGT (niPGT): Science and Methodologies

Noninvasive Preimplantation Genetic Testing (niPGT) is an evident paradigm shift from the traditional invasive biopsy-based methods (PGT-A). It analyzes cell-free DNA (cfDNA) released by the embryo into its culture environment. Through this, niPGT’s main objective is to assess embryonic chromosomal status without physically compromising the embryo. This segment of the article aims to understand its scientific basis and methodologies, aspects that are crucial for evaluating its potential and limitations.

### 5.1. Sources of Cell-Free DNA: Blastocoel Fluid vs. Spent Culture Medium

The cfDNA analyzed in niPGT originates from embryonic cells undergoing apoptosis or other forms of cellular activity during in vitro undertakings. Balstocoel fluid (BF) and spent culture medium (SCM) are analyzed as the two primary sources.

BF refers to the fluid filling the blastocoel cavity of the blastocyst, usually at days 5–6. BF’s DNA is presumed to be primarily embryonic in origin (90–95%), emanating from dying cells within the enclosed cavity [9,59,60,61,62,71]. BF’s advantages include the potentially higher concentration of embryonic DNA. The fluid also has less direct exposure to external contaminants and is more representative of the inner cell mass (ICM) based on the underpinnings of the cavity formation dynamics [59,60,71]. A significant disadvantage is that BF is challenging in terms of technicality, that is, aspirating without damaging the blastocyst. It also requires skilled micromanipulation similar to a biopsy. Further, the aspiration in itself is a form of intervention, despite being less invasive than cell removal. On top of these, not all blastocysts have easily aspirable fluid, and the potential for DNA degradation is high if processing is not initiated as soon as possible [59,60,61,71].

SCM is the culture medium in which the embryo has been grown. It is typically collected after blastocyst culture, usually between days 5 and 6. cfDNA in SCM is released by the embryo’s trophectoderm cells into the surrounding environment [2,11,60,63,64,65,66]. SCM’s key advantage is that it is a true non-invasive DNA collection method. Hence, it significantly limits any mechanical stress on the embryo. It is also simple to perform since only medium aspiration is required. With this, parallel testing of multiple embryos is possible, and logistically, it integrates seamlessly into standard IVF lab workflows [2,9,60,63,66,67,68,71]. Its main weakness compared to BF is that the concentration of embryonic cfDNA is very low (10–40%) and the risk of contamination from maternal DNA or other external sources is fairly high [2,11,61,69]. Linked to these factors are the facts that the DNA is likely to be highly fragmented and, due to the external influences, prone to degradation. Further, the dilution effect from the medium volume is substantial, underlying low DNA concentrations. Additionally, unlike BF, DNA may originate primarily from TE, thus less representative of the ICM [2,9,11,59,60,62,63,64,65,67,70,71].

Of the 2 primary sources, SCM is the predominant alternative for niPGT research and development. This is due to its practical advantages and truly noninvasive nature, albeit the significant weaknesses of low DNA yield and contamination [2,9,60,63,66,67,68,69,70,71].

### 5.2. Extraction, Amplification, and Sequencing Methods

The workflow for niPGT from SCM involves highly sensitive techniques. These are necessary to handle the minimal amount and already degraded cfDNA. The first step is sample collection and preparation. During this step, SCM is carefully aspirated after the embryo has been removed, all while ensuring minimal disturbance [9,11,63,66,67,69,71]. Precautionary measures taken to minimize contamination include rigorously removing cumulus cells before fertilization and the use of sequential media that is changed at specific times, such as before blastulation [9,11,63,67,69]. To prevent DNA damage, immediate processing is fostered [11,60,64,71]. After the sample has been collected and prepared, cfDNA is then extracted. This step entails using specialized kits designed for lower concentration, fragmented cfDNA, which exhibits similarity to liquid biopsy techniques [9,11,59,60,62,64,67]. The objective at this step is to maximize the recovery of the lowly concentrated embryonic DNA while minimizing contaminants. Efficiency is necessary at this step [11,60,64,71].

To maximize potential DNA yield, WGA is conducted. It is an essential step due to the extremely low quantity of embryonic cfDNA. However, while necessary, it presents key operational challenges. For instance, WGA introduces significant technical noise. It also exhibits amplification bias, allele dropout, and the introduction of aneuploidy signals [59,60,62,64,65,71]. Regardless, there is room for optimization. For instance, as noted by Hu et al. (2023), this can be achieved by comparing different WGA kits and protocols to improve fidelity and reduce artifacts [62,64]. Some protocols excel at amplifying specific chromosomes to reduce complexity [72].

With the DNA extracted, the next step is genetic analysis, also described as sequencing or detection. This is achieved by next-generation sequencing (NGS), the dominant platform for niPGT-A. NGS excels in simultaneously sequencing millions of DNA fragments [2,9,59,60,61,62,65,66,67,68,69,70]. There are numerous ways it can achieve this. The first approach is low-pass whole genome sequencing (LP-WGS). This sequences the entire genome at low coverage. With that, it is sufficient to detect whole-chromosome aneuploidies, specifically by analyzing sequence read counts mapped to each chromosome. As per evidence, niPGT-A leverages this approach the most [2,59,60,65,67,70,71]. There is also higher coverage WGS, also referred to as targeted NGS. This is less common compared to LP-WGS because it is costly and computationally complex. However, it still has its place, specifically being used to detect segmental imbalances or mosaicism [65]. Besides NGS, there is quantitative polymerase chain reaction (qPCR). It operates by using fluorescent probes to quantify the number of copies of specific chromosomal regions [72]. Compared to NGS, it is faster, less expensive, and easier to use due to the simple data analysis. Unlike NGS, it is limited to predefined chromosomal targets. It also has lower resolution and is less effective for detecting mosaicism or partial aneuploidies. Further, it is more susceptible to amplification bias and contamination artifacts [65,72].

The last step after DNA sequencing and detection is bioinformatics analysis. This step is a sophisticated computerized approach required to analyze NGS data [2,59,60,65,67,70]. Key tasks entail genome mapping, calculating chromosomal ratios, and aneuploidy identification through relevant statistical models [2,59,60,65,67,70].

### 5.3. Current Techniques

Several niPGT techniques are in use. However, niPGT for aneuploidy (niPGT-A) is the most common. It is the most developed application. Its primary focus is on detecting whole-chromosome aneuploidies using LP-WGS of cfDNA from SCM [2,61,62,65,66,67,68,70]. Within niPGT-A, NGS, specifically LP-WGS, is the gold standard due to its genome-wide scope and scalability [2,60,62,65,67,69,70]. There are also emerging applications, mainly niPGT for monogenic disorders (niPGT-M) and structural rearrangements (niPGT-SR). However, these still face significant operational limitations due to the complexities that must first be overcome, such as the need for structural variant detection from minute, fragmented DNA [67,72].

### 5.4. Challenges

Despite its promise, niPGT, just like the invasive PGT it is trying to replace, faces significant technical hurdles. The first key concern revolves around maternal DNA contamination. The contaminating DNA is primarily from maternal cumulus and granulosa cells surrounding the oocyte, residual sperm cells, and sometimes, even endometrial cells. It is often present in much higher quantities than embryonic cfDNA in SCM, thus fostering dilution [2,9,11,59,60,61,62,63,64,65,66,67,68,69,70]. The consequence of maternal DNA’s presence is that it can completely mask the embryonic signal. This often results in false euploid calls or false aneuploid calls in cases where the contaminating cells are aneuploid [9,63,70,71]. A key mitigation measure for this challenge is rigorous oocyte denudation [9,11,63]. Other relevant operational measures include the careful handling of media, changing medium after cumulus removal or early cleavage stages [11,63,69], and adopting ways that can selectively digest maternal DNA to avoid pertinent contamination [63].

The second key challenge linked to contamination issues is low cfDNA yield and quality. As significantly highlighted by relevant evidence, the amount of embryonic cfDNA released into SCM is extremely low. It is also highly variable between embryos [11,59,60,61,64,65,70,71]. Additionally, embryonic cfDNA tends to be highly fragmented. This further complicates analysis and amplification [59,60,71]. The key consequence is that all these result in high rates of amplification failure or poor-quality WGA. The outcome is usually no result (NR) or inconclusive results [11,59,60,64,70,71]. Other consequences of the low yield and poor quality are the limitation in detecting mosaicism and segmental imbalances, as well as compromises on the robustness and reliability of niPGT [59,60,65,70]. A key way to mitigate this challenge, as per the literature, is to optimize culture conditions to potentially enhance DNA release [11,69]. Technicians can also leverage larger starting medium volumes [11,71] and enhance the amplification process [72].

The third key challenge limiting this test’s efficiency is inter-embryo variability. Biologically, embryos of similar morphology and development stage are highly likely to exhibit variances in the amount, timing, and pattern of cfDNA release. Key factors underlying this phenomenon include embryo quality, metabolic activity, and genetic status [11,59,60,61,70,71]. The key consequence of this challenge is that it significantly hampers standardization, making universal diagnostic thresholds hard to establish. The variability also underlies inconsistent performance, as well as discordance rates with TE biopsy [59,60,61,65,70,71]. To mitigate the variability, amplification protocols need to be continually refined, embryo-specific normalization factors need to be developed, and more studies conducted to fully conceptualize the biological determinants of release [59,60,67].

### 5.5. Summary

niPGT primarily uses cfDNA from spent culture medium analyzed via NGS following WGA. It is a significant noninvasive alternative to biopsy. The core scientific premise banks on using naturally released embryonic DNA [2,60,67,71]. However, its technical efficiency is significantly limited by substantial maternal DNA contamination that masks the embryonic signal, the severely low and variable embryonic cfDNA that underlies high failure rates, and inter-embryo biological and technical variability that negatively impacts standardization [9,11,59,60,61,62,63,64,65,70,71].

## 6. Clinical Evidence: Can niPGT Replace Biopsy?

The potential of niPGT fully replacing TE biopsy and becoming the standard method for aneuploidy screening fully depends on robust clinical validation. Current evidence, derived from key studies and meta-analyses comparing niPGT to TE biopsy-based PGT-A, reveals significant promise. However, this promise is marred by substantial limitations, more so regarding reliability and consistency.

### 6.1. Key Studies and Meta-Analyses

Xu et al. [73] provided the seminal proof-of-concept for niPGT-A. In their study, they demonstrated that cell-free DNA (cfDNA) in spent culture medium (SCM) could reflect the embryonic ploidy status. The concordance was validated by comparing the SCM results to the chromosomal analysis of the corresponding whole donated embryo, not a biopsy, reporting a sensitivity of 88.2% and specificity of 84.0% for detecting abnormalities. The study also reported the detection of aneuploidy and suggested the potential for the identification of mosaicism. This foundational study was crucial for igniting the field, while also acknowledging key challenges such as the low quantity of embryonic DNA and the risk of contamination.

Huang et al. [58] provide validation and optimism. This study reported a high concordance of approximately 80% between niPGT-A (SCM) and TE biopsy. The scholars even went ahead to claim that niPGT-A might be more reliable than biopsy in some cases. This is due to better representation of mosaic status [58]. The assertions significantly boost optimism about replacing biopsy.

In their pivotal prospective multicenter study, Rubio et al. [74] explore the concepts of fertility and sterility. This is the first large-scale (n = 1301 blastocysts), prospective, multicenter trial using niPGT-A (SCM) for embryo selection. Pertinent findings indicated a high concordance rate with TE biopsy of approximately 85% for full chromosome aneuploidies. Further, the results also showed the promising ongoing pregnancy rates to be 50% after the intervention of euploid niPGT-A transfers [74]. This study has been crucial in moving niPGT-A towards clinical application. However, it also acknowledged a significant rate of no result (NR) and discordance. These are attributed to maternal contamination and biological variability.

Subsequent Validation and Discordance Studies:

Huang et al. [75] validated a targeted NGS niPGT-A method. They showed high sensitivity and specificity. However, they also noted and acknowledged technical challenges [75]. Shitara et al. [71] found that cfDNA in SCM reflected chromosomal status. This was after an extended culture. Nevertheless, they also noted variability compared to TE biopsy [71]. Sakkas et al. [69] demonstrated that implementing a specific niPGT-A culture protocol can potentially impact viability and outcomes. This excels in highlighting the sensitivity of the method to procedural scrutiny [69].

Studies Highlighting Limitations:

Hanson et al. [76] reported alarmingly high rates of DNA amplification failure at approximately 50%. They also indicated the poor correlation between niPGT-A (SCM) and TE biopsy results. These findings significantly challenge the reliability of niPGT-A, thus substantially countering the initial bout of optimism [76]. Volovsky et al. [70] critically assert that despite the promise of niPGT-A being real, current evidence shows otherwise. For instance, it has lower sensitivity and specificity than TE biopsy. niPGT-A also suffers from high failure rates and significantly lacks validation for clinical use as a biopsy replacement [70].

Meta-Analyses:

Huang et al. [77] included 20 studies in their meta-analysis. Their findings supported the conclusion that niPGT-A demonstrates good diagnostic performance in research settings. However, they also emphasized significant heterogeneity and the influence of technical factors. Overall pooled sensitivity was indicated as 0.84 and specificity as 0.85 compared to TE biopsy [77]. What these figures insinuate is the lack of near-perfect accuracy that is sometimes claimed. Li Piani [78] primarily focus on biopsy harm. As such, they significantly highlight the need for a reliable non-invasive alternative. With this assertion, they indirectly insinuate that the current niPGT-A is yet to be proven to efficiently fulfill that role [78].

### 6.2. Sensitivity and Specificity Compared to PGT-A

The metrics vary. However, they excel in indicating that niPGT-A is yet to be fully considered an equivalent. Regarding sensitivity, values range widely across studies, from approximately 50% to over 90% [58,71,73,74,75,76,77]. Pooled specificity estimates by Huang et al. [77] were around 0.83–0.90, which, while higher than sensitivity, is still suboptimal. These findings imply that niPGT-A misses a significant proportion of aneuploid embryos, hence false negatives. Contributors to this limitation include low embryonic DNA concentration, contamination and dilution by maternal DNA, amplification failure, and biological sampling variances evident in SCM and TE cells [70,71,76,77].

Regarding specificity, findings also show variability. However, it is generally higher than sensitivity, often reported between 80 and 95%, but still slightly lower compared to TE biopsy [58,71,74,75,76,77]. Pooled estimates by Huang et al. [77] are around 0.83–0.90. The key contributors to the false positives include dilution by maternal DNA, WGA bias, and a significant emphasis on noisy data [70,71,76,77].

On the concordance rate, which tends to be reported instead of the traditional sensitivity and specificity, the value is indicated to be above 80% by high-impact studies [58,74]. However, real-world data and meta-analyses show substantial discordance of 15–30% or more. In this case, niPGT-A is performing worse than biopsy in head-to-head comparisons for diagnostic reliability [52,70,76,77]. Regarding the no-result (NR) rate, it is often reported to range from 10 to 50% [69,70,74,76]. The implications of this is that the embryo will have to be discarded, a rescue biopsy performed, thus defeating the whole purpose of noninvasiveness, or transfer the embryo blindly [52,70,74].

### 6.3. Real-World Success Rates in RPL/RIF Subpopulations

Data on niPGT-A outcomes in RPL and RIF patients is significantly limited and less encouraging compared to the initial broad population studies highlighting its potential efficiency. Firstly, no targeted RCTs are comparing live birth rates after niPGT-A selection, TE biopsy selection, and morphology-based selection in RPL/RIF populations [35,52,70,79]. Secondly, the high rate of low sensitivity is significantly concerning. This is because undetected aneuploid embryos can be transferred, increasing the risk of miscarriage [35,70,77]. Thirdly, the high NR rate is a major issue for RIF/RPL patients, more so for those having fewer embryos. It is a major disadvantage to discard or blindly transfer embryos without genetic information due to test failure [52,70,74]. In their analysis, Volovsky et al. [70] argue that the data from Rubio et al. [74] actually showed lower ongoing pregnancy rates with niPGT-A selection compared to conventional PGT-A selection in their cohort. However, it is worth noting that Rubio et al. [74] included patients with previous failures, and subgroup analysis specific to confirmed RIF/RPL was not the study’s primary focus, thus limiting any significant conclusions.

In a different analysis, Guan et al. [42] focus on an RCT in young RIF patients that compared morphology alone against PGT-A (biopsy). The RCT did not directly test niPGT. However, it showed no CLBR benefit for PGT-A over morphology alone in the young RIF group [42]. The findings raise valid concerns regarding the ability of any form of PGT-A, especially the less reliable niPGT-A, to offer a significant advantage for young RIF over standard care. This indirectly insinuates that niPGT-A might fail to meaningfully benefit patients. Overall, validation is inadequate in the high-risk groups to make definitive conclusions on niPGT-A’s efficacy in RPL/RIF [35,70]. Hence, subgroup analysis is needed to effectively determine real-world success.

### 6.4. Cases Where niPGT Changed Clinical Management

Despite limitations, niPGT-A has shown potential utility in specific scenarios. One of them is rescue for biopsy failure or inconclusive results. In cases where TE biopsy fails by exhibiting outcomes such as no result or inconclusive diagnosis, niPGT-A on SCM has been indicated to have the potential to provide a genetic assessment. It offers information that would otherwise not be available, thus enabling the transfer of an embryo that would have been discarded [9,74,80,81]. In their study that provided this evidence, Li et al. [80] explored this specifically for mosaic embryos. Another scenario where niPGT excels is detecting uniform aneuploidy (whole-chromosome aneuploidy). Despite confidence levels varying, niPGT-A has the potential to clearly identify uniform aneuploidy in cases of high confidence calls. Through such, it prevents the transfer of embryos with very low potential, just like biopsy [58,73,74]. niPGT also excels in avoiding biopsy risks. Granted that reliability is confirmed, it thrives on its non-invasiveness [81]. Lastly, in the research context, niPGT-A allows repeated sampling of the same embryo’s environment at different time points. This offers unique research insights into embryonic biology and DNA release dynamics that a biopsy cannot provide [71].

### 6.5. Summary

Current clinical evidence indicates that niPGT-A cannot yet reliably replace TE biopsy as the standard method for PGT-A. Landmark studies like Huang et al. [75] and Rubio et al. [74] demonstrate feasibility and potential. However, subsequent research and critical reviews reveal significant limitations. These include variable and often suboptimal sensitivity and specificity compared to biopsy, a significantly high rate of no result, and susceptibility to errors from maternal contamination and low DNA yield. Further, evidence for its effectiveness in improving live birth rates for the target RPL and RIF subpopulations is lacking. Indirect data suggest that it is significantly limited [35,42,70]. Overall, niPGT-A can be leveraged in rescuing biopsy failures, as a back-up, and as a research tool [9]. However, its inconsistency and diagnostic uncertainty prevent it from being the clinically validated substitute for TE biopsy, remaining an adjunct at best. To address the gaps, more evidence from large-scale studies and well-designed RCTs in RPL and RIF populations is needed.

## 7. Application in RPL/RIF: Hope or Hype?

The potential of niPGT presents a significant promise for patients and clinicians facing the harsh and heartbreaking realities of RPL and RIF. All the publicity emanates from its capability to revolutionize embryo selection noninvasively. However, translating this promise into reliable clinical practice, more so for RIF and RPL patients, remains a challenging course. The current application of niPGT in RPL/RIF sits precariously between hopeful innovation and premature hype.

### 7.1. Benefits: The Foundations of Hope

niPGT’s key advantage and selling point is its non-invasiveness. It eliminates the need for physically removing cells. As such, this preserves embryo viability and developmental potential. This is mainly achieved by avoiding mechanical stress and potential damage to the ICM [55,65,82,83]. Through non-invasiveness, niPGT addresses a core ethical and biological concern of invasive PGT-A. This is particularly important when dealing with precious embryos from patients with limited ovarian reserve, a common scenario in RPL/RIF [25,55,65,82]. Linked to non-invasiveness is the aspect of embryo preservation. By avoiding biopsy, niPGT eliminates the need for concurrent vitrification of the biopsied embryo. This eliminates the stress associated with double handling and allows embryos to be transferred fresh as required or vitrified intact [10,55,65,82]. This is particularly more appealing in RIF cases where endometrial receptivity timing is crucial [25,84]. Thirdly, niPGT has high patient acceptability. Many patients prefer it due to reduced anxiety about biopsy-related embryo harm [1,55,82]. Patients are also ethically comforted by the fact that this procedure avoids embryo manipulation [55,65,82]. It is also a simplified process compared to invasive biopsy [1,82], thus improving the patient’s experience in an already stressful journey [1,25,84]. Additionally, it also has the potential for wide access. If proven reliable and cost-effective, the simpler workflow would make genetic testing accessible to more patients. This would include some RPL/RIF cases previously deemed unsuitable for PGT-A [82].

### 7.2. Drawbacks: The Reality Check

Inconsistency and unreliability are the most significant issues hindering niPGTs’ clinical adoption, especially for RPL/RIF, where diagnostic accuracy is paramount [54,55,65,83]. One key issue established in the earlier review of literature is the high rate of no result (NR). Reported in 10–50% of the cases, amplification failure underlies niPGT’s inability to provide a reliable genetic analysis [10,55,83]. This is more devastating for RPL/RIF patients with few embryos, since viable embryos can be discarded or blindly transferred [55]. Another reliability issue is that niPGT’s accuracy is variable. This is evidenced by several studies highlighting niPGT’s suboptimal sensitivity and specificity compared to TE biopsy [54,65,77,83]. False negatives are more impactful for RPL patients given the high risk of miscarriage. Contrastingly, false positives are more detrimental for RIF patients with limited embryos [55,65,83]. niPGT also exhibits high discordance; its results frequently disagree with TE biopsy. This undermines confidence in its ability to accurately reflect the embryo’s true chromosomal status [54,65,83]

Furthermore, besides reliability and inconsistency, niPGT-A faces significant regulatory skepticism. It is not yet approved for routine clinical use by major bodies like the FDA or EMA, which classify it as an investigational technology [54,55,65]. This stance is strongly supported by professional guidelines. For instance, the ESHRE Good Practice Recommendations explicitly advise that non-invasive PGT-A should be considered experimental due to the lack of robust evidence demonstrating its efficacy and safety [85]. This consensus, rooted in concerns over insufficient clinical validation, unreliable diagnostic accuracy, and the absence of standardized protocols, currently limits widespread clinical implementation and insurance coverage for niPGT-A [54,55]

Another key drawback is the lack of protocol standardization. For instance, the variance in culture conditions such as media type, volume, and exposure significantly impacts cfDNA quantity and quality [10,65]. Sample collection and handling, as well as DNA processing, also vary between labs, directly contributing to inconsistent results [54,55,65].

There is also the issue of unproven clinical utility in RPL/RIF. This is highlighted by the lack of robust evidence from well-designed RCTs demonstrating niPGT-A’s efficiency over traditional methods in RPL or RIF populations [48,55,83].

### 7.3. How IVF Centers Are Currently Using or Trialing niPGT

Clinical application is cautious and primarily experimental due to current limitations in evidence. niPGT is presently more leveraged in research and validation protocols [10,54,55,83]. For instance, Cheng et al. [10] describe a protocol for a double-blinded RCT (NCT05622288) that seeks to compare niPGT-A to morphology alone. Such a trial is essential, but the results are pending [10]. It is also being leveraged as a backup for specific scenarios. For instance, it is being used as a rescue for biopsy failure, providing information where none was available [65,80]. It can be used when patients specifically prefer it. Individuals receive extensive counseling, more so about niPGT’s limitations, such as the risk of false negatives/positives and NR rates [55,65,82]. It is also being used as an adjunct to TE biopsy and morphokinetic assessment [65]. Lastly, it is being leveraged for general infertility rather than a targeted first-line solution for RPL/RIF due to the higher risks involved [55]. Despite these various applications, there lacks widespread adoption. Many IVF centers are cautious about niPGT’s unresolved technical and validation issues, particularly when it comes to the welfare of RPL/RIF patients [54,55,65].

### 7.4. Integration with Time-Lapse, Morphology, and AI

niPGT’s efficiency and full potential lie in integration with other advanced embryo assessment tools. Amalgamated, they will create a multi-modal diagnostic approach. These tools include time-lapse imaging (TLI), which functions by providing continuous data on embryo morphokinetics [86,87]. Pertinent Integration aims to understand the dynamics of DNA shedding in relation to developmental events and embryo quality markers [86]. It also targets to leverage pertinent morphokinetic parameters to predict which embryos are more likely to yield successful niPGT results [86,87]. In their overview, Oh et al. [86] explore nucleolar dynamics as a potential marker. Additionally, integrating niPGT with TLI can enhance interpretation through holistic embryo assessment [87]. With morphology, pertinent insights such as blastocyst grade alongside available niPGT results and TLI data can refine selection algorithms. This has the potential to mitigate the impact of niPGT’s false results or NRs [65,87].

Integration with AI is crucial for several aspects. One of them is bioinformatics analysis. With effective AI algorithms, technicians can better distinguish true embryonic aneuploidy signals from background noise such as maternal DNA contamination in NGS data. This improves diagnostic accuracy [65,82,83]. Further, AI integration underlies predictive modeling. There are numerous crucial datasets such as TLI, morphology, patient history, cfDNA characteristics, fragmentation patterns, and hormonal profiles. Integrating all of them via AI can predict embryo viability and ploidy status more accurately than any single method [65,82,83,87]. For instance, Stankewicz [87] conceptualized optimizing IVF by controlling for both aneuploidy and receptivity using testing. This is a goal where integrated AI could play a role. AI can also be leveraged for complex scenarios. Managing and interpreting the vast, multi-dimensional data from various sources, such as TLI and niPGT, requires powerful AI [65,82].

### 7.5. Hope or Hype

niPGT offers a compelling vision for the future of embryo selection in RPL and RIF. It is truly noninvasive, embryo-preserving, and significantly more patient-centered [1,65,82]. The theoretical benefits are substantial and have driven ongoing research [10,54]. However, the current reality leans heavily towards its status as just hype. That is because it is not ready in any significant manner for routine clinical application in these challenging populations [54,55,65,83]. Key issues preventing it from reliably replacing invasive PGT-A include inconsistency, unreliability, lack of standardization, and regulatory skepticism. The most crucial of them all is the absence of proven clinical benefit in improving live birth rates for RPL/RIF [48,55,83].

Some centers are already exploring its use in specific well-evaluated scenarios that include application in research protocols [10,55]. However, it is not the standard of care. The integration of niPGT with TLI, advanced morphology, and AI represents the most promising pathway forward. They have the potential to overcome current limitations by creating a more robust, multi-parameter assessment system [65,82,83,86,87]. However, until these integrations become effective and rigorous evidence is available demonstrating clear efficacy and reliability in RIF/RPL, niPGT ought to be approached with caution and managed expectations. Presently, it just remains a promising tool under intense development, its application in managing RPL and RIF residing firmly in the realm of hope rather than established clinical practice [54,55,65,82,83].

## 8. Future Perspectives and Clinical Recommendations

### 8.1. Need for Prospective, Multicenter RCTs

Current studies are largely retrospective or underpowered. Hence, there is a significant need for prospective, multicenter RCTs that compare LBR in niPGT-A versus TE-PGT-A or morphology-based selection. These trials must prioritize RPL/RIF subpopulations. This is where diagnostic accuracy impacts outcomes most significantly [48,55]. The focus of the pertinent RCTs should be on clinical utility, such as the ability of niPGT-A reducing miscarriage rate, failure mitigation, and long-term outcomes, particularly on child health and development [48,55,88].

### 8.2. Combining niPGT with Epigenetic and Metabolomic Profiling

The future of niPGT-A may lie not in standalone genetic analysis. Rather, there is a chance of its integration with other ‘omics’ data from the spent culture medium to create a holistic viability score. This could involve:

- Epigenetic Profiling: This entails analyzing cell-free DNA methylation patterns in SCM to assess embryonic epigenetic stability. Such is crucial for implantation and fetal development [1,88].
- Metabolomic Profiling: This entails assessing the flux of metabolites such as amino acids like glutamate in SCM. Such can reflect embryo viability and mitochondrial function. It has also been correlated with ploidy status [1,2].

A synergistic diagnostic model that combines niPGT-A with such epigenomic and metabolomic biomarkers could potentially outperform genetic screening alone. This is more particular in cases of idiopathic RPL/RIF [1,48].

### 8.3. Standardization of Protocols and Reporting

Critical gaps in niPGT workflows highlight the urgent need for consensus, as highlighted in Table 4 below.

### 8.4. Tailored Approaches for Patient Subgroups

Risk-stratified niPGT applications are necessary. In AMA and DOR, technicians need to prioritize niPGT-A given the higher aneuploidy burden. However, they must validate against TE-biopsy due to the high risk of false negatives [48,55]. In unexplained RPL, they should combine niPGT-A with endometrial immune profiling and thrombophilia panels. This is fundamental to addressing multifactorial etiology [1,89]. In young RIF patients, niPGT-A should be limited to research purposes only until more evidence is available confirming utility [48]. Ethically, niPGT-A should be offered as a biopsy-free option after thorough counseling on its potential limitations, such as the risk of false negatives, which is critical for RPL [55].

## 9. Conclusions: A Roadmap for Responsible Innovation

niPGT holds transformative potential. However, it requires rigorous validation before replacing TE biopsy. Until prospective RCTs confirm efficiency over TE-PGT-A, niPGT-A should remain investigational for RPL/RIF management. Its greatest value and potential lies in rescue testing for biopsy failures. It also has the ability to thrive as a research tool in multi-parameter embryo selection models.

### 9.1. Reiterating the Promise and the Prerequisites

NiPGT’s potential is undeniable and compelling. Its core advantage is noninvasiveness, which addresses fundamental ethical and biological concerns associated with TE biopsy. This is achieved through preserving embryo integrity and eliminating procedural risks. Through its strengths, it fosters enhanced patient acceptability, reducing potential negative psychological outcomes linked with embryo manipulation. Further, niPGT optimizes embryo preservation and allows flexibility in transfer timing. Research about its development is ongoing, and this is premised on the core attributes of safety, efficiency, and patient-friendly genetic assessment.

### 9.2. Confronting the Current Reality: Barriers to Clinical Adoption

niPGT’s transition from promise to proven practice faces formidable hurdles. One key issue is the unresolved technical limitations. This is evidenced by the core issues of maternal DNA contamination, critically low and variable embryonic cfDNA yield, and significant inter-embryo biological variability. These aspects fundamentally compromise the reliability and consistency of results. They contribute to unacceptably high rates of test failure and diagnostic discordance compared to the traditional gold standard (TE biopsy). The second key issue is insufficient and inconclusive clinical evidence. Currently, there are no well-designed, large-scale RCTs demonstrating the efficiency of niPGT-A in improving LBRs or reducing miscarriage rates specifically in RPL or RIF patients compared to the conventional methods. The variable sensitivity and specificity fall short of the diagnostic accuracy required for routine clinical reliance in RIF/RPL. In fact, evidence suggests its performance in these specific populations may be suboptimal.

The third key issue is the lack of standardization and regulatory hurdles. This underlies inconsistent results across labs. It is based on this that reliability issues persist, informing regulatory skepticism that has seen niPGT being relegated to investigational purposes only, as per expert recommendations such as ESHRE guidelines. The last aspect is its unproven utility in complex etiologies. 

### 9.3. Imperative Call for Cautious Integration and Further Research

The path forward demands cautious integration and intensified research. Firstly, rigorous RCTs comparing its outcomes with the current standards of TLI and TE PGT-A are needed. Secondly, standardization is needed to foster and guarantee consistency, comparability, and data quality. Thirdly, key stakeholders should explore multi-omic integration, as well as TLI and AI-driven predictive modeling to enhance viability assessment. Fourthly, its application needs to be tailored and cautious, being leveraged only for certain selected scenarios. Specifically, it needs to be used as an adjunct and implemented mostly in favorable cases.

Overall, as niPGT shines as a beacon of hope for a less invasive future in embryo selection, its light is currently too dim to effectively guide clinical decision-making for the complex and emotionally draining cases of RIF and RPL. Widespread adoption requires not just optimism. Instead, concrete evidence from rigorous research, standardized methodologies, and proven efficacy in the specific populations it aims to serve are needed. Hence, evidence-based integration, not haste or hope, needs to define the next chapter of niPGT development.

## Figures and Tables

**Table 1:** Clinical Overview of Recurrent Pregnancy Loss (RPL) and Recurrent Implantation Failure (RIF).

Aspect	Recurrent Pregnancy Loss (RPL)	Recurrent Implantation Failure (RIF)
**Definition**	Refers to 2 two or more consecutive pregnancy losses before 20 weeks of gestation. Some guidelines, such as ESHRE, define it as 3 or more losses [1,2,12].	Lack of universal consensus; typically defined as ≥3 failed IVF cycles with high-quality embryo transfers [13,14].
**Incidence**	Affects 1–5% of reproductive-aged women [2,15].	Affects 10–15% of IVF patients [13,16]. Incidence is rising for both conditions due to delayed childbearing and increased ART use [15,17].
**Common Etiologies**		
**Anatomic**	Uterine anomalies such as a septate uterus and adhesions account for 10–15% of cases [13,18].	Endometrial polyps and submucosal fibroids disrupt implantation [14,15].
**Endocrine**	Thyroid dysfunction (TSH > 2.5 mIU/L), diabetes, and prolactinemia contribute to 17–25% of cases [13,19].	Luteal phase deficiency and polycystic ovary syndrome (PCOS) are key contributors [20,21].
**Immunological**	• Dysregulated uterine NK (uNK) cells, aberrant cytokine profiles (e.g., increased IFN-γ, decreased IL-10), and elevated uNK cytotoxicity [19,22,23,24]. • Autoimmunity: Antiphospholipid syndrome (APS) causes 15% of cases via thrombotic placental injury [7,25,26]. • Alloimmune rejection: Maternal-fetal HLA compatibility (e.g., shared paternal HLA-C alleles) and Treg deficiencies disrupt tolerance [18,27,28,29].	• Dysregulated uNK cells and aberrant cytokine profiles are also implicated [19,22,23,24]. • Endometrial inflammation, characterized by overexpression of TNF-α and IL-6, is associated with RIF [14,30].
**Thrombophilic**	• Heritable: Factor V Leiden and prothrombin mutation increase miscarriage risk 3–5 times [26,29]. • Acquired: Anti-β2-glycoprotein antibodies cause placental infarction [25,26].	(Thrombophilic factors are more directly linked to post-implantation loss in RPL, but may contribute to the implantation environment).
**Genetic**	• Parental: Karyotype abnormalities account for 2–5% of RPL [31,32]. • Embryonic: Aneuploidy drives >50% of miscarriages, which worsens with advancing maternal age [31,32].	Embryonic aneuploidy is also a significant factor; It is the main cause.

**Table 2:** Types and Clinical Impact of Embryonic Aneuploidy.

Type of Chromosomal Abnormality	Definition	Clinical Impact/Prevalence	Key References
**Whole-Chromosome Aneuploidy**	The gain (trisomy—Trisomy 16) or loss (monosomy—Monosomy X) of entire chromosomes.	Responsible for more than 90% of embryonic losses.	[35,36]
**Polyploidy**	The presence of extra full sets of chromosomes (e.g., triploidy—an equivalent of 69 chromosomes).	A distinct category of numerical abnormality often leading to early miscarriage.	-
**Segmental Aneuploidy**	Partial deletions or duplications of chromosomal segments (e.g., 22q11.2 microdeletion).	Implicated in 5–10% of miscarriages.	[4,37]
**Complex Aneuploidy**	Multiple chromosomal errors within a single embryo.	More prevalent in advanced maternal age.	[35,38]
**Mosaicism**	The presence of two or more cell lines with different chromosomal complements within the same embryo.	Clinical impact varies significantly based on the type and proportion of abnormal cells. However, it is a major challenge for diagnostic accuracy.	[4,39,40]
**General Context**	Origin: Errors during meiosis (gamete formation) or mitosis (early embryonic cell division). Overall Effect: Disrupts normal embryonic development, leading to implantation failure or early miscarriage. Risk Factor: The risk increases exponentially with advancing maternal age due to an age-related decline in oocyte quality and chromosomal segregation fidelity.		[4,35,36,38,40,41,42,43,44,45,46]

**Table 3:** Comparison of Current Embryo Selection Strategies for Aneuploidy.

Strategy	Method/Principle	Key Benefits/Reported Accuracy	Major Limitations	Key References
**Morphokinetic Grading**	Assessment of blastocyst morphology (e.g., Gardner’s criteria) and dynamic development via time-lapse imaging (TLI).	Non-invasive. Serves as the foundational assessment in IVF. ~65% accuracy in predicting euploidy.	Poor correlation with chromosomal status, that is, high-grade morphology embryos can be aneuploid and vice versa. Lacks sufficient sensitivity and specificity.	[4,35,40,43,44,48,49]
**Invasive PGT-A (Gold Standard)**	Trophectoderm (TE) biopsy (5–10 cells) on Day 5/6, followed by genetic analysis (e.g., Next-Generation Sequencing—NGS).	Direct assessment of chromosomal status. Increases live birth rate (LBR) per transfer by 20–30% in RIF/RPL. Reduces miscarriage rates per transfer.	Invasive; risk of embryo damage. Diagnostic errors due to mosaicism (false positives/negatives). High cost, logistical complexity, and ethical concerns over discarding embryos.	[3,4,6,35,36,37,38,39,40,41,42,43,44,45,46,47,48,49,50,51,52,53,54,55,56,57]
**Non-invasive PGT-A (niPGT-A) (Investigational)**	Analysis of cell-free DNA (cfDNA) released into spent blastocyst culture medium (SCM).	Completely non-invasive—preserves embryo integrity. High patient acceptability. Potential for wider access. 70–90% concordance with TE biopsy in studies.	Inconsistent accuracy—variable sensitivity (70–85%) and specificity (88–92%). High rate of amplification failure (10–50%). Vulnerable to maternal DNA contamination and low DNA yield. Not yet validated for RPL/RIF.	[9,11,42,58,59,60,61,62,63,64,65,66,67,68,69,70]

**Table 4:** niPGT’s standardization requirements for the technology’s improvement. For reporting frameworks, stakeholders should implement STARD-NiPGT guidelines to ensure transparency in sensitivity and specificity metrics and NR rates [55].

**Domain**	**Standardization Requirements**
Culture conditions	Media type/volume, change intervals, incubation duration [2,55]
Sample handling	SCM collection timing, storage, contamination controls [55]
Analytical methods	WGA kits, sequencing depth, bioinformatics [2,55]

## Data Availability

No new data were created or analyzed in this study. All data discussed in this paper are derived from previously published studies, which are properly cited within the manuscript.

## Abbreviations

**Abbreviation**	**Full Form**
**ADO**	Allele Dropout
**AI**	Artificial Intelligence
**AMA**	Advanced Maternal Age
**AMH**	Anti-Müllerian Hormone
**ANXA5**	Annexin A5
**APS**	Antiphospholipid Syndrome
**ART**	Assisted Reproductive Technology
**BF**	Blastocoel Fluid
**cfDNA**	Cell-Free DNA
**CLBR**	Cumulative Live Birth Rate
**DOR**	Diminished Ovarian Reserve
**EQC**	External Quality Controls
**ESHRE**	European Society of Human Reproduction and Embryology
**HLA**	Human Leukocyte Antigen
**ICM**	Inner Cell Mass
**IR**	Implantation Rate
**ISO**	International Organization for Standardization
**LBR**	Live Birth Rate
**LP-WGS**	Low-Pass Whole Genome Sequencing
**MDA**	Multiple Displacement Amplification
**NGS**	Next-Generation Sequencing
**niPGT**	Noninvasive Preimplantation Genetic Testing
**niPGT-A**	Noninvasive Preimplantation Genetic Testing for Aneuploidy
**niPGT-M**	Noninvasive Preimplantation Genetic Testing for Monogenic disorders
**niPGT-SR**	Noninvasive Preimplantation Genetic Testing for Structural Rearrangements
**NK cells (uNKs)**	Natural Killer cells (Uterine Natural Killer cells)
**NR**	No Result
**PCOS**	Polycystic Ovary Syndrome
**PGT**	Preimplantation Genetic Testing
**PGT-A**	Preimplantation Genetic Testing for Aneuploidy
**PGT-M**	Preimplantation Genetic Testing for Monogenic disorders
**PGT-SR**	Preimplantation Genetic Testing for Structural Rearrangements
**PPV/NPV**	Positive Predictive Value/Negative Predictive Value
**qPCR**	Quantitative Polymerase Chain Reaction
**RCT**	Randomized Controlled Trial
**RIF**	Recurrent Implantation Failure
**RPL**	Recurrent Pregnancy Loss
**SCM**	Spent Culture Medium
**SET**	Single Embryo Transfer
**STARD-PGT**	Standards for Reporting Diagnostic Accuracy Studies-PGT
**STR**	Short Tandem Repeat
**TE**	Trophectoderm
**TLI**	Time-Lapse Imaging
**Treg**	Regulatory T cell
**TSH**	Thyroid-Stimulating Hormone
**WGA**	Whole Genome Amplification
**WGS**	Whole Genome Sequencing
